# New Perspectives on Ebola Virus Evolution

**DOI:** 10.1371/journal.pone.0160410

**Published:** 2016-08-01

**Authors:** Celeste J. Brown, Caleb J. Quates, Christopher A. Mirabzadeh, Craig R. Miller, Holly A. Wichman, Tanya A. Miura, F. Marty Ytreberg

**Affiliations:** 1 Department of Biological Sciences, University of Idaho, Moscow, Idaho, United States of America; 2 Institute for Bioinformatics and Evolutionary Studies, University of Idaho, Moscow, Idaho, United States of America; 3 Center for Modeling Complex Interactions, University of Idaho, Moscow, Idaho, United States of America; 4 Department of Physics, University of Idaho, Moscow, Idaho, United States of America; Metabiota, UNITED STATES

## Abstract

Since the recent devastating outbreak of Ebola virus disease in western Africa, there has been significant effort to understand the evolution of the deadly virus that caused the outbreak. There has been a considerable investment in sequencing Ebola virus (EBOV) isolates, and the results paint an important picture of how the virus has spread in western Africa. EBOV evolution cannot be understood outside the context of previous outbreaks, however. We have focused this study on the evolution of the EBOV glycoprotein gene (GP) because one of its products, the spike glycoprotein (GP_1,2_), is central to the host immune response and because it contains a large amount of the phylogenetic signal for this virus. We inferred the maximum likelihood phylogeny of 96 nonredundant GP gene sequences representing each of the outbreaks since 1976 up to the end of 2014. We tested for positive selection and considered the placement of adaptive amino acid substitutions along the phylogeny and within the protein structure of GP_1,2_. We conclude that: 1) the common practice of rooting the phylogeny of EBOV between the first known outbreak in 1976 and the next outbreak in 1995 provides a misleading view of EBOV evolution that ignores the fact that there is a non-human EBOV host between outbreaks; 2) the N-terminus of GP_1_ may be constrained from evolving in response to the host immune system by the highly expressed, secreted glycoprotein, which is encoded by the same region of the GP gene; 3) although the mucin-like domain of GP_1_ is essential for EBOV *in vivo*, it evolves rapidly without losing its twin functions: providing *O*-linked glycosylation sites and a flexible surface.

## Introduction

Small but devastating outbreaks of Ebola virus disease (EVD) have occurred in humans since the mid-1970s, but the recent outbreak in the western Africa countries of Guinea, Sierra Leone and Liberia dwarfed all recorded human cases with 28,640 total cases and 11,316 deaths as of February 17, 2016 (http://www.cdc.gov/vhf/ebola/outbreaks/2014-west-africa/case-counts.html). This recent outbreak was caused by Ebola virus (EBOV), which has also been detected in humans in various parts of central Africa in multiple distinct outbreaks between 1976 and 2014. EBOV has also been detected in wild animals, including chimpanzees and gorillas [1]. While infectious virus has not been isolated from bats, they are generally considered the most likely reservoir species for EBOV between human outbreaks [2, 3]. Intermittent outbreaks and an unknown non-human reservoir have made it difficult to track EBOV evolution [4].

The glycoprotein gene (GP) is a good candidate for understanding the EBOV evolutionary response to host interactions because the protein it encodes, the spike glycoprotein (GP_1,2_), directly interacts with the host immune system. Sera from human patients during the convalescent phase of EVD have a high prevalence of GP_1,2_-specific antibodies [5]. Furthermore, EBOV vaccines and therapeutic antibodies are being developed that target GP_1,2_ [6, 7]. The GP-encoded protein is cleaved to produce GP_1_ and GP_2_ [8]; and the mature spike glycoprotein GP_1,2_ is composed of trimers of these two subunits [9]. Multiple GP_1,2_ proteins extend from the viral membrane and are responsible for host cell attachment and fusion (reviewed in [10]). The C-terminal half of GP_1_ is a mucin-like domain that is heavily glycosylated with both *N*-linked and *O*-linked glycans [11]. This domain interacts with molecules on the cell surface, possibly lectins, inducing endosome formation by the cell [12–15]. Once the virus is within the endosome, the C-terminus of GP_1_ is cleaved to release the glycan cap and the mucin-like domain [16]. This exposes the receptor binding site, so that the cleaved GP_1_ can bind to its receptor, currently believed to be a lysosomal cholesterol transporter, NPC1 [17, 18]. GP_1_ is then released so that GP_2_ can create a fusion pore that allows viral entry into the cytoplasm [19].

GP also encodes two other proteins, secreted GP (sGP) and small, secreted GP (ssGP) [20, 21]. Transcripts for ssGP are so rare that we do not consider its role further in this study [22]. The sGP protein may be playing an important role in GP evolution that has not been explored previously. The GP transcript is produced when the viral RNA polymerase slips and introduces an extra adenosine in a seven-nucleotide, polyA stretch; the sGP transcript is formed when transcriptional slippage does not occur [20, 21]. Thus, the sGP protein is encoded by the 5-prime half of GP and is out of frame with the GP_1_ protein for the last 67 amino acids of its sequence. Unlike GP_1_, sGP is a homodimer and therefore has a different quaternary structure and may have a different tertiary structure than GP_1_ [23]. The sGP transcript is predominant in *in vivo* EBOV infections, composing 80–99% of transcripts [22, 24]. In animal and cell line passaging experiments, there is strong selection to maintain transcripts for sGP in animals but not in cells [24]. The sGP protein has antigenic properties and is thought to act as a decoy for the host immune system [25]. Thus both GP_1,2_ and sGP are important targets for understanding EBOV evolution.

Phylogenetic analyses conducted during or after each EVD outbreak have produced conflicting results. Until multiple outbreaks in 2001–05, the analyses indicated that a single evolving isolate was responsible for each successive outbreak, spreading EVD in a wave-like pattern from east to west [26]. During the 2001–05 outbreaks along the border between Gabon and the Republic of Congo (RC), significant effort was made to find the zoonotic source of the virus, because this epidemic may have been causing high mortality among non-human primates as well as humans [27, 28]. Two unrelated EBOV isolates were sequenced from this effort with non-human primates carrying isolates similar to one of the human isolates [28]. Short cDNA sequences from several different genera of bats suggested that each had been infected by isolates closely related to one of the human EBOV isolates [2, 29]. The isolate that caused the 2007–08 outbreak in the Democratic Republic of Congo was also quite different from the isolate that had been evolving and spreading since 1976 [30]. Initial analysis from early in the western Africa outbreak indicated that this epidemic was coming from an isolate that was also distinct from the 1976 lineage [31]. This historical perspective has been lost in recent analyses of the current outbreak, in which the root of the phylogeny is placed between the 1976 and 1995 outbreaks in the Democratic Republic of Congo [32]. The placement of the root at this point obfuscates important aspects of EBOV evolution [4].

Understanding the evolution of EBOV is essential for determining how this virus is maintained between outbreaks, how it then emerges to wreak such havoc in human populations, and how outbreaks might be curtailed in the future [4, 22, 31–35]. We have concentrated our efforts on the GP gene to understand EBOV evolution for two reasons: first, there are more GP sequences available than any other gene sequence; and second, the proteins it encodes interact directly with the host immune system and therefore are expected to evolve by positive selection. Indeed, one to five codons within GP have been inferred to have evolved by positive selection [36–38]. In this study, we have taken a closer look at adaptive evolution in GP by focusing on the details of where multiple substitutions at single amino acid positions are occurring in the context of both the structure of the glycoprotein and the phylogenetic relationships amongst the isolates. In particular, the majority of substitutions are in the mucin-like domain, a heavily glycosylated region [10], which is structurally disordered. Disordered protein regions often evolve more rapidly than the structured regions of the same protein [39]. The large number of substitutions in this region might at first appear to indicate neutral evolution [36], but here we consider these substitutions in terms of where they have arisen in EBOV's evolutionary history.

Our study focused on the evolution of the EBOV glycoprotein gene with particular emphasis on adaptive evolution, thus producing a meaningful phylogeny was integral to this work. We analyzed both GP and sGP gene sequences from the first recorded outbreak in 1976 through the western Africa outbreak up to the end of 2014. Multiple reports have been published on the western Africa outbreak; here we concentrated on EBOV evolution prior to 2014 in the context of the current outbreak, rather than on the 2013–2016 outbreak *per se*. Based upon an unrooted phylogeny of EBOV, we show that the numbers of substitutions at both unique sites and sites at which multiple substitutions arose are greater in the disordered regions of GP than in the structured regions; evidence for positive selection is greatest in this disordered domain; host switching may have affected EBOV evolution during the putative non-human primate outbreak; the sGP gene shows the same pattern of substitutions in the short, sGP-unique domain that is predicted to be disordered, but otherwise seems to evolve at a single, relatively slow rate. We discuss these findings in terms of the role GP and sGP play in evading the host immune system.

## Results

### Phylogenetic relationships among isolates from all EVD outbreaks based upon GP

Ninety-six unique nucleotide sequences for the glycoprotein gene (GP) from EBOV were used to infer the phylogenetic relationships among isolates from different outbreaks (accession numbers in S1 Fig). These sequences included those collected from human outbreaks, from gorillas and chimpanzees during outbreaks in the early 2000s [28], and from humans whose infections were traced to infected gorillas at the same time [27]. There are also sequences from the outbreak in western Africa sampled prior to January 2015 and two isolates from Liberia sampled in 2015 (see S1 Fig for GenBank accession numbers). There is one sequence that was isolated after multiple passages in a mouse.

**Fig 1:**
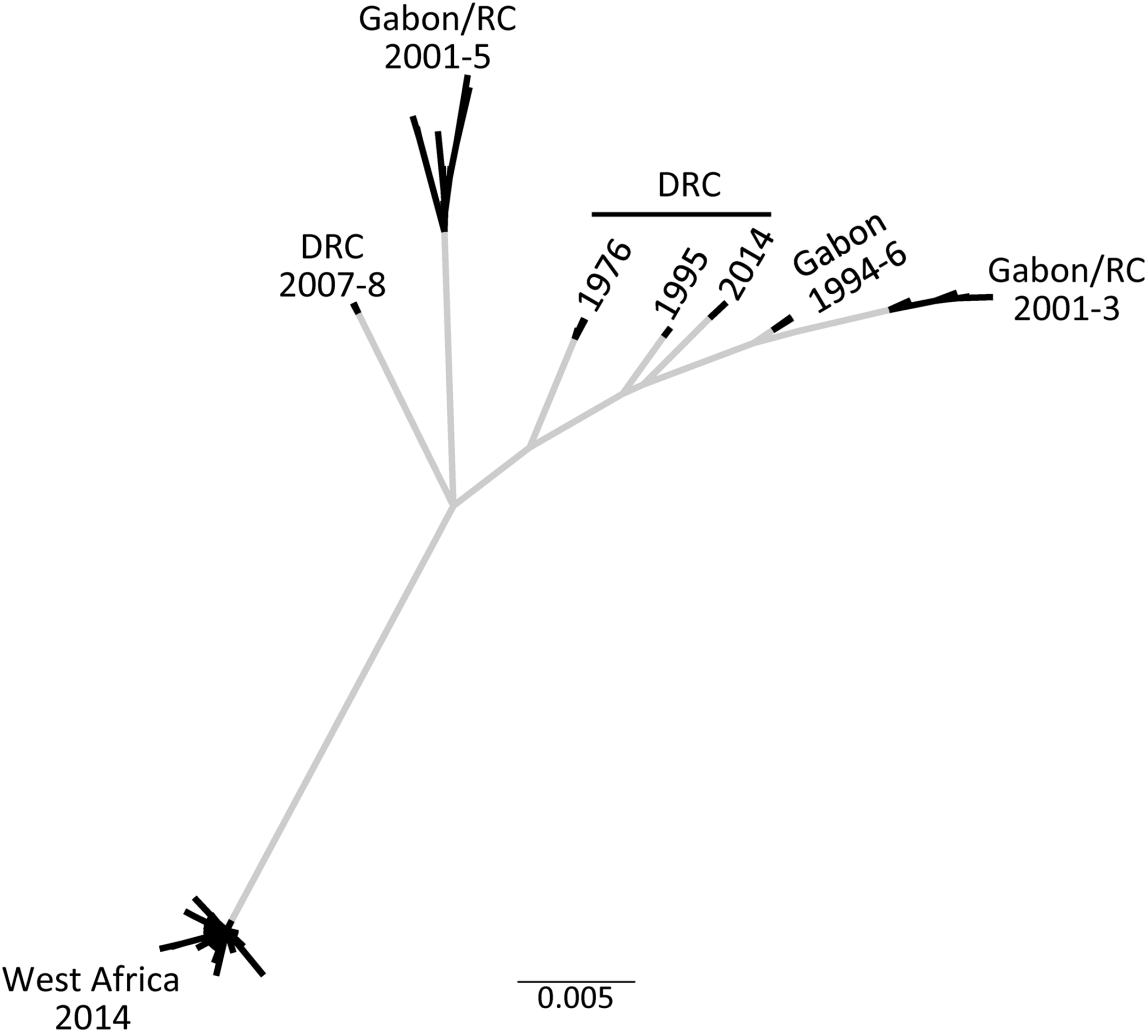
Unrooted phylogeny of EBOV GP gene emphasizing unknown evolution within the natural host. Grey lines indicate evolution is occurring within the natural host prior to the outbreak, while black lines indicate evolution within the outbreaks. For clarity, labels for sequences have been removed and replaced with labels for location and year of sampling (DRC: Democratic Republic of Congo, called Zaire from 1971 to 1997; RC: Republic of Congo; West Africa includes Guinea, Sierra Leone and Liberia). Scale bar indicates number of substitutions per site.

The phylogenetic relationships among these 96 GP sequences are depicted as an unrooted tree in Fig 1. There are distinct branches for this unrooted tree for several outbreaks: 1) in the DRC in 2007–08; 2) in western Africa in 2014; and 3) in one set of samples from the Gabon/RC outbreak in 2001–05. The fourth branch includes the outbreaks in the DRC (Zaire) in 1976, 1995 and 2014, in Gabon in 1994–96, and a second set of samples from the Gabon/RC region from 2001–03.

### Models of GP evolution

Using the phylogenetic tree in Fig 1, we tested for variable rates of evolution at each codon of the aligned gene sequences, specifically testing whether some codons are evolving at a faster rate than others. The best fitting model for the complete GP sequence had three rates, and 2% of the 676 codons were estimated to have omega values significantly greater than 1, suggesting that positive selection has been important in the evolution of this gene (Table 1).

**Table 1:** Best models of codon sequence evolution and their estimates of dN:dS (ω) for the EBOV GP gene.

Sequences	Best Model ^a^	ω (proportion of sites)^b^		
		ω<1^c^	ω = 1^d^	ω>1^e^
GP Complete	M2a Positive Selection	0.20 (74%)	1.0 (24%)	6.2 (2%)
GP Structured	M1a Nearly Neutral	0.00 (74%)	1.0 (26%)	
GP Disordered	M2a Positive Selection	0.68 (96%)	1.0 (3%)	13 (1%)
sGP	M0 One Rate	0.27 (100%)		

^a^ model code and description of model from PAML

^b^ percentages indicate the proportion of sites inferred to have these dN:dS ratios.

^c^ values near zero indicate purifying selection, values near 1 indicate relaxed selection

^d^ indicates neutral evolution

^e^ indicates positive selection

**Fig 2:**
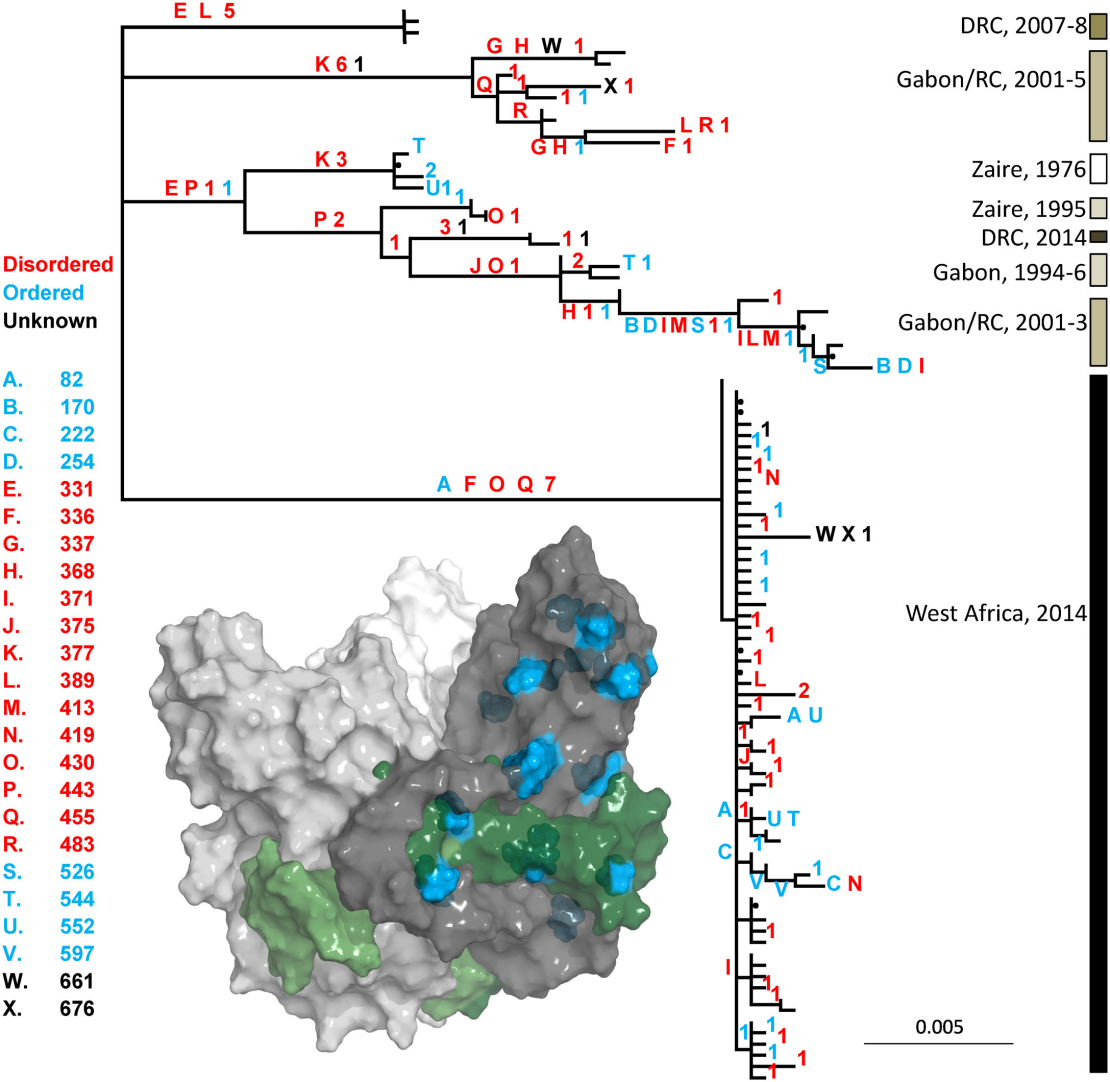
Unrooted phylogeny for EBOV glycoprotein gene showing all nonsynonymous substitutions. Substitutions are colored by location in disordered (red) or structured (blue) regions. Numbers indicate counts of unique substitutions that occurred only on one branch. Letters indicate sites (inset legend) at which substitutions were found on more than one branch. Dots indicate branches of zero length. Bars on right indicate years and locations of disease outbreaks and are shaded by date of outbreak. (DRC: Democratic Republic of Congo, called Zaire from 1971 to 1997; RC: Republic of Congo; West Africa includes Guinea, Sierra Leone and Liberia) Scale bar indicates number of substitutions per site. Inset is molecular structure of EBOV GP_1,2_ΔmucΔtm with shading to highlight each GP_1,2_ dimer and the GP_2_ subunit in shades of green (based on PDB: 3CSY; [9]). Amino acids colored blue in the structure show sites where substitutions arose only once coinciding with the numbers on the phylogeny.

Because it is well known that disordered regions of proteins may evolve more rapidly than the structured regions of the same protein [39], we performed the same evolutionary analysis as was done for GP on the codons for the structured and disordered regions, separately. Along with the disordered mucin-like domain, there are several small regions that are disordered in the crystal structure of GP_1,2_ [9]. Fig 2 is a reformatted version of Fig 1; it shows where amino acid substitutions were inferred to occur during evolution given that the ancestor is at the base of the four main branches. The number of nonsynonymous (amino acid altering) substitutions that occurred only once and the sites where multiple nonsynonymous substitutions were found are colored in Fig 2 to highlight those changes that arose in structured (blue) vs disordered (red) regions of GP. Fig 2 illustrates the greater number of substitutions in the disordered regions than in the structured regions. The best fitting model for the structured regions indicated that they are evolving at a nearly neutral rate (Table 1) such that three quarters of the codons were invariant, and the other 25% were evolving at rates indistinguishable from neutrality (ω = 1). Substitutions that arose only once in the structured regions are highlighted in blue on the GP_1,2_ structure (Fig 2). The disordered regions, on the other hand, were best fit by a model under relaxed selection (ω≈ 0.7) or neutrality (ω = 1) for almost all of the sites, with 1% of sites evolving in response to positive selection. Thus this gene is showing a characteristic rapid rate of evolution in the disordered region, and there is no strong evidence for positive selection in the structured regions.

**Fig 3:**
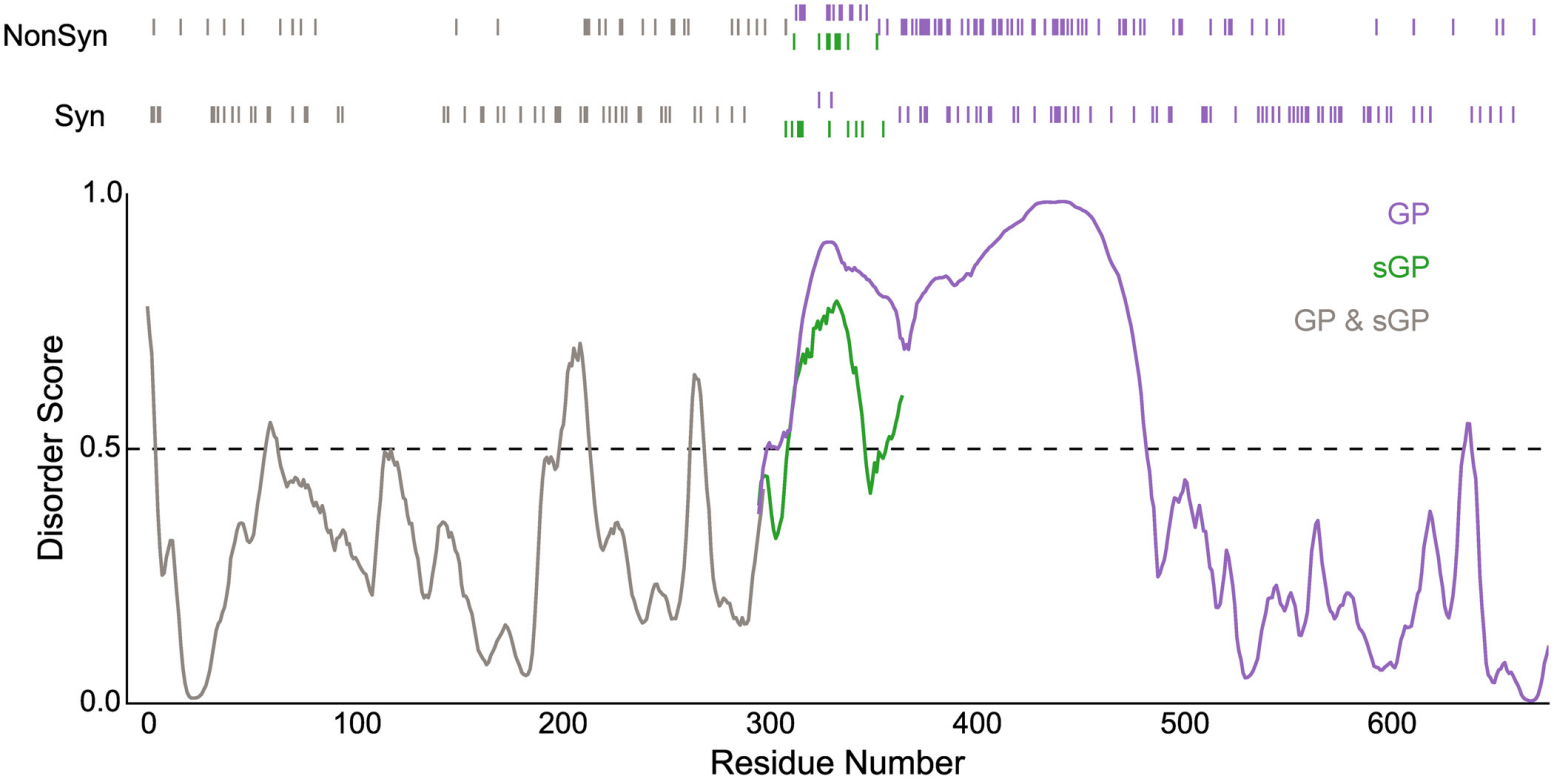
Rapid rates of amino acid sequence evolution are associated with disordered domains. Nonsynonymous and synonymous substitutions aligned with PONDR-VSL2 disorder scores for glycoprotein (GP; purple) and secreted glycoprotein (sGP; green). Overlapping region where GP and sGP have the same sequence is shown in gray. Scores greater than 0.5 indicate predicted disorder.

Relaxed purifying selection in the disordered regions and strong purifying selection in the structured regions are further illustrated by observing the codons at which nonsynonymous and synonymous substitutions are occurring (Fig 3). The bottom graph shows the disorder scores for the entire GP amino acid sequence (grey and purple) estimated using the VSL2 disordered protein predictor. Regions inferred to be disordered have scores greater than 0.5, and these regions align well with the known disordered regions. The bars above this graph indicate codons in which nonsynonymous and synonymous substitutions have occurred. Nonsynonymous substitutions are sparse relative to synonymous substitutions in the regions upstream and downstream of the disordered mucin-like domain (residues 313–501). Conversely, there are more nonsynonymous substitutions than synonymous substitutions in the disordered regions. The difference in proportion of synonymous and nonsynonymous substitutions among the disordered, structured and unknown structure regions is significant (Fisher's Exact Test: χ ^2^ = 23; p-value = 0.0002). On average, approximately 25% of nucleotide positions within a protein coding region are synonymous because they do not alter the amino acid sequence, and 75% of sites are nonsynonymous. In the disordered region of GP_1_, 34% of substitutions are synonymous and 66% are nonsynonymous. Thus, the disordered regions are accumulating nonsynonymous substitutions at a rate approaching that expected by chance, indicating these regions are generally evolving under very relaxed selection.

### Further evidence for positive selection in GP

**Fig 4:**
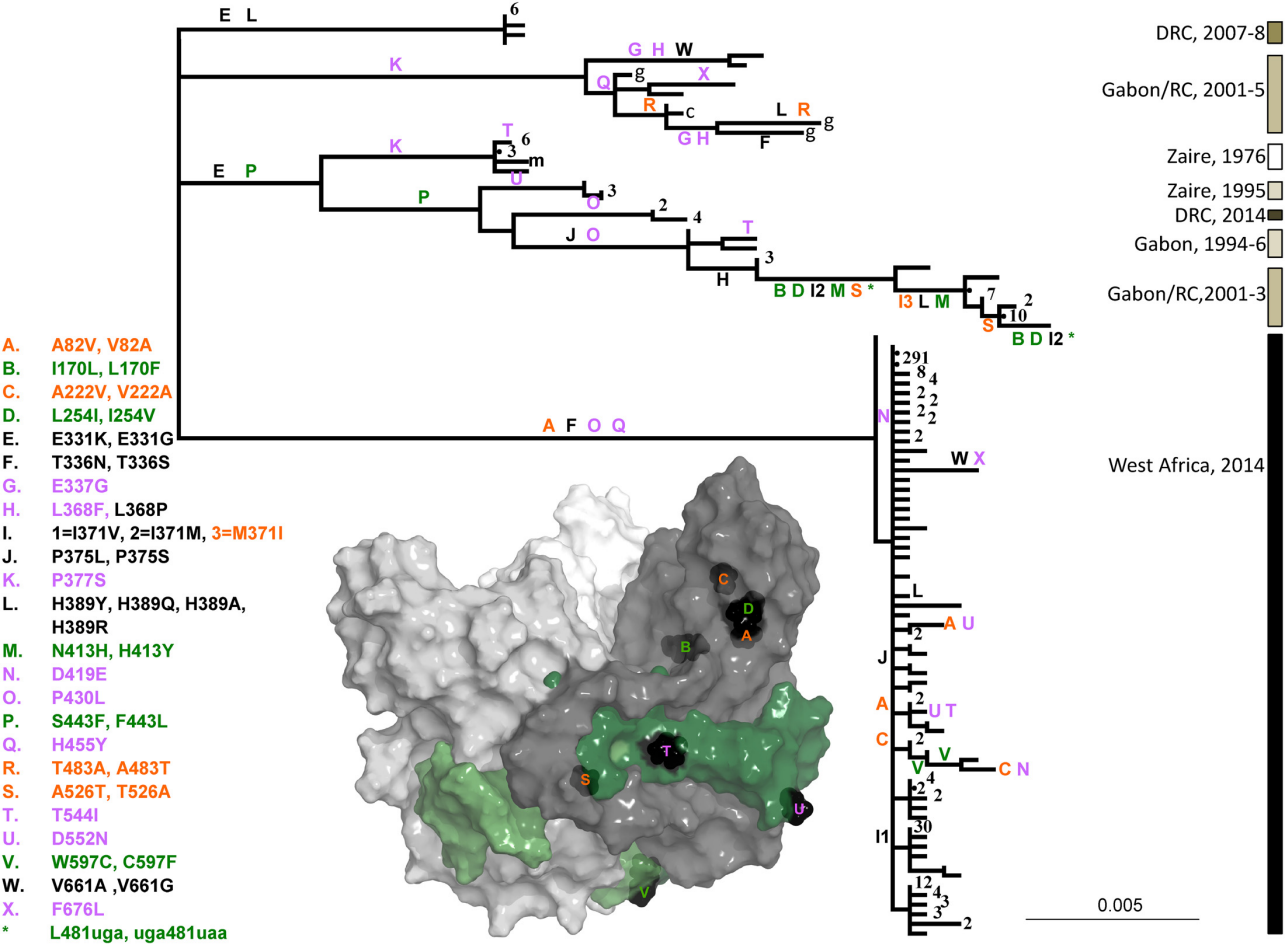
Unrooted phylogeny for EBOV glycoprotein gene showing sites experiencing more than one nonsynonymous substitution. Codons at which more than one nonsynonymous substitution arose are colored to indicate types of multiple mutations: parallel (purple), reversion (orange), different mutant amino acids (black) and sequential changes (green). Key on the left shows substitutions (from amino acid, site, to amino acid), and their order of occurrence on the tree is from top left to bottom right, unless indicated with numbers. Numbers at tips indicate number of sequences greater than one that had the same nucleotide sequence. Dots indicate branches of zero length. Lowercase g, c and m indicate samples from gorilla, chimpanzee and mouse adaptation experiments, respectively. Bars on right indicate years and locations of disease outbreaks and are shaded by date of outbreak. (DRC: Democratic Republic of Congo, called Zaire from 1971 to 1997; RC: Republic of Congo; West Africa includes Guinea, Sierra Leone and Liberia) Scale bar indicates number of substitutions per site. Inset is molecular structure of EBOV GP_1,2_ΔmucΔtm complex with shading to highlight each GP_1,2_ dimer and the GP_2_ subunit in shades of green (based on PDB: 3CSY; [9]). Black amino acids on structure indicate sites of substitutions.

Further evidence for positive selection comes from looking more carefully at the amino acid substitutions that have arisen in the glycoprotein over all outbreaks. The overall evolutionary model inferred by PAML indicated that there were approximately 15 sites evolving under positive selection. Codons 389 and 430 had posterior probabilities great than 95% that they were subject to positive selection. The histidine at position 389 mutated to four different amino acids; two of these are on internal branches of the phylogeny indicating that they were not subject to purifying selection (Fig 4; L). The proline at position 430 mutated to leucine on three occasions, and two of these are on internal branches (Fig 4; O). There are, however, other codons that have undergone repeated substitutions, a hallmark of positive selection, and these are also highlighted in Fig 4. As indicated by the different colors in the figure, there are 24 codons at which more than one nonsynonymous mutation occurred: eight of these codons mutated more than once to the same amino acid, seven codons mutated more than once to different amino acids, five mutated to one amino acid that then mutated to a third, and four codons underwent back mutations. For 144 total amino acid substitutions out of 676 codons, we would expect to see only 15 randomly chosen sites that have undergone two substitutions, and only one site to have undergone three, thus substitutions at these sites appear to be nonrandom. Note that many of these positively-selected sites were identified previously for data collected prior to the western Africa outbreak using the random effects likelihood method [37]; the inclusion of sequences from 2014 during the western Africa outbreak extends this list of sites.

Others have noted that EBOV growth in cell culture can promote evolution of GP, particularly at the poly U tract that leads to the transcriptional stutter [10, 27]. Most samples taken prior to the outbreak in western Africa were amplified in tissue culture for variable, and in some cases unknown, numbers of passages prior to sequencing. In addition, three samples from the recent outbreak in western Africa (S1 Fig; KP096420, KP096421, KP096422) were also amplified prior to sequencing. It is possible that some of the parallel evolution that we see is due to adaptation to this novel environment. For example, substitutions from threonine to isoleucine at position 544 (Fig 4; T) and from aspartic acid to asparagine at 552 (Fig 4; U) are consistent with parallel evolution in tissue culture.

There is also strong evidence that host-switching has played a role in the evolution of GP aside from the evolution within the unknown, long term host. From 2001–05 there were outbreaks of EVD along the border between Gabon and the Republic of Congo. These outbreaks may have affected non-human primates as well as humans as evidenced by severe declines in gorilla and chimpanzee populations and the presence of EBOV cRNA in tissue samples [27]. Although a direct causal chain was not proven, several human infections were associated with handling diseased gorillas and chimpanzees [27, 28]. Interestingly, this outbreak involved at least two isolates of EBOV (Figs 1, 2 and 4). Multiple substitutions are hallmarks of these two branches of the phylogeny. There are 0.49 substitutions per isolate as compared with 0.22 for the 1994–96 outbreak in Gabon and 0.036 in the current outbreak in western Africa. These multiple substitutions take the form of reversions at three sites (Fig 4: I, R, S), step-wise substitutions to new amino acids at three sites (Fig 4: B, D, M), parallel substitutions at three sites (Fig 4: G, H, X), and substitutions to different amino acids than those found within (Fig 4: H, L) and outside of these outbreaks (Fig 4: F, I, W). Thus the apparent switch between the reservoir host, non-human primates and humans appears to have resulted in positive selection in these outbreaks.

### Models of sGP evolution

The gene that encodes the glycoprotein also encodes two other proteins. The transcript that is found in the greatest abundance encodes the secreted glycoprotein (sGP), which is encoded by the gene sequence without transcriptional slippage and is half the length of GP [25]. After the point where slippage occurs to produce the spike glycoprotein, the sequence of sGP diverges [20]. Because overlapping genes often affect one another's evolution, a phylogenetic analysis of sequences encoding pre-sGP was also performed, and the evolutionary rates for this coding sequence were estimated. Fig 3 shows that the sGP gene sequence has fewer nonsynonymous and more synonymous substitutions than GP in the divergent reading frame (Fisher's Exact Test: χ ^2^ = 8.1; p-value<0.007), although the number of nonsynonymous substitutions in the disordered region of sGP is still high relative to the rest of sGP. Because the one rate model so rarely explains the DNA sequence evolution of a protein-coding gene, it is surprising that the best fitting model for the sGP gene is a single rate model (Table 1).

## Discussion

Although inferring a species phylogeny based upon a single gene can be misleading, the GP gene appears to carry most of the phylogenetic signal in the EBOV genome [33]. While our results may differ in minor details from whole genome phylogenies, the overall view of EBOV evolution is consistent with previous work. Importantly, multiple GP sequences are available for all of the outbreaks, providing a more complete picture of EBOV evolution. When unrooted phylogenies are compared, this picture is the same across all previous analyses of whole genomes or GP alone.

From our unrooted phylogenetic analysis and those of others (Fig 1; Gire, et al. 2014), we infer that there were different zoonotic sources for the four main branches of the tree. There appear to be different sources for the two isolates in the Gabon/Republic of Congo region in 2001–05, for the isolate in the Democratic Republic of Congo (Zaire) in 2007–08 and for the 2013–2016 outbreak in western Africa. Additionally, the following outbreaks appear to have derived from the same zoonotic source: the DRC (Zaire) in 1976, 1995 and 2014, Gabon in 1994–96 and one isolate from the Gabon/RC 2001–05 outbreak. The original papers that presented EBOV GP or genome sequences from Gabon/RC 2001–05, DRC 2007–08 and western Africa 2014 mentioned that each of these outbreaks appear to be coming from a different source than the first outbreak in 1976 [28, 30, 31].

After the initial description of the western Africa outbreak [31], the phylogenies of EBOV isolates have used the initial outbreak in 1976 as the root [22, 32–35], even when this placement leads to "puzzling" results [4]. On the other hand, all phylogenies that included ebolaviruses other than EBOV, root the tree elsewhere and inconsistently, but not between the 1976 and 1995 outbreaks [31, 37]. We suspect that whatever the host reservoir is, the true root lies within the genealogical history of that population (or community), because humans are likely an evolutionary dead end for EBOV between outbreaks [40].

Our analysis reveals another significant gap in our understanding of EBOV evolution: what accounts for differences in EBOV between outbreaks? While there are clearly different ancestors for most outbreaks, it is not known how much of that variation is due to population or species differences between reservoir hosts, and how much, if any, is due to evolutionary changes that fix in the viral population at the beginning of each new outbreak in humans and other primates. This important question can only be addresses by examining EBOV variation in bats, but the only sequences available are short stretches of the viral polymerase L gene from the 2001–05 outbreak in the Gabon/RC region [2, 29]. These sequences varied both within and between the three species of bats from which they were taken [2]. When sequences from ebolaviruses other than EBOV are included in the phylogenetic analysis, the bat sequences are basal to the EBOV outbreaks from 1976 to 2005 [2]. Results using these short sequences suggest that accounting for evolution within the reservoir host is essential for understanding EBOV evolution leading to human outbreaks.

Although the spike and secreted glycoproteins interact with the immune system, the structured regions do not show a strong evolutionary response to immunological pressure (Table 1). GP_1_ performs several functions that might be adversely affected by amino acid substitutions: receptor binding and association and disassociation with GP_2_. We propose that the secreted protein, sGP may buffer the N-terminus of GP_1_ from evolving in response to the immune system. GP_1,2_ is a prominent target for antibodies from EBOV survivors, and most antibodies also bind to sGP [5, 41]. In fact, antibodies that bind to both GP_1,2_ and sGP react more strongly with sGP, suggesting that sGP induced their production [41]. Furthermore, the production of sGP is conserved *in vivo*, but not *in vitro* where there are no antibodies [24, 42]. The consequence of sGP acting as an antibody decoy may be that the pressure on the N-terminus of GP_1_ to evolve in response to immune evasion is greatly reduced. This is supported by the sparse number of nonsynonomous mutations in GP_1_ upstream of the mucin-like domain (Fig 3). The low sequence divergence of GP_2_, on the other hand, may be due to constraints imposed by its essential function in cell entry.

In contrast, evolution in the disordered regions of EBOV suggests a strong response to the host immune system (Table 1; Fig 4). The disordered mucin-like domain provides low rates of neutralization by antibodies and high rates of antigenic change without adversely affecting the functions of highly antigenic sites in GP_1_ [43]. First, the domain is heavily glycosylated by the host cell, thus presenting a signature that the host defines as "self" [44]. Because the domain is inherently flexible, a hallmark of protein disorder, it may move to mask potential epitopes. In addition, flexibility may present an ever-changing landscape to the immune system. Second, when there are few structural constraints on disordered proteins, they are free to evolve under relaxed purifying selection [45]. It was recently shown that evolution in the mucin-like domain does not affect the prediction of intrinsic disorder in this region [36]. Thus, the mucin-like domain may be evolving to escape detection within the reservoir host and during human infections. This form of positive selection may appear to be neutral at the DNA sequence level because it does not lead to repeated changes at particular amino acids. Sequence changes across the entire domain, however, may still be due to selection for immune evasion. It seems quite probable that the disordered mucin-like domain is found in this, and many other viruses, because it can evolve rapidly without losing its function.

Although the 2013–16 outbreak in western Africa provides a unique opportunity to study the evolution of EBOV during the course of an outbreak, it does not shed much light on curtailing future outbreaks. Here we examine evolution both within and between outbreaks. By looking at the evolution of the protein that has the most interaction with the host immune system, we have shown that EBOV is subject to both positive selection and relaxed purifying selection in the mucin-like domain and to strong purifying selection in the N-terminus of GP_1_. Our study highlights the need for a better understanding of evolution in the reservoir host. Differentiating between evolution in the reservoir host and at the initiation of an outbreak may be critical for developing strategies to recognize and inhibit future outbreaks.

## Materials and Methods

### Sources of sequences

Aligned nucleotide sequences encoding the Ebola virus glycoprotein gene were downloaded from the NCBI Virus Variation website on 5/13/15. Additional sequences from [27] were extracted directly from Genbank and partial sequences were removed. Identical nucleotide sequences were reduced to a single representative sequence using the ElimDupes website (hcv.lanl.gov/content/sequence/ELIMDUPES/elimdupes.html). The sequence of the secreted glycoprotein sGP was translated directly from this reduced set of sequences. To produce the pre-GP protein sequence, an A was inserted in the 7 A stretch to simulate transcript slippage that leads to the spike glycoprotein; a small subset of isolates has a T in this stretch, and an A was also added to this region to produce the pre-GP protein sequence. Subregions of the nucleotide sequences were extracted from the alignment in order to perform separate phylogenetic and evolutionary analyses on regions that are known to be structured (nt 100–570, 643–837, 898–933, 1510–1803), disordered (nt 571–642, 838–897, 934–1509, 1804–1902) or whose structures are unknown (1–99, 1903–2034) based upon the GP structure (PDB ID: 3CSY; [46]). Note that the structured region does not include either the signaling peptide or the transmembrane domain; the sGP gene sequence, on the other hand includes the signal peptide.

### Phylogenetic inference and analysis of adaptive molecular evolution

Each set of gene alignments: complete sGP, complete GP, structured GP, and disordered GP, were subjected to the following phylogenetic analysis. Using the DT-ModSel algorithm, the best-fitting model of DNA sequence evolution was inferred for each alignment. These models were then used in the PAUP* 4.10 [47] program to infer the maximum likelihood phylogeny for each alignment. Each alignment and unrooted phylogeny were then used in PAML 4.7 [48] to detect nonsynonymous and synonymous substitutions and to infer positive selection among codons using five models: a single omega (dN/dS ratio) value for the whole alignment (M0); a neutral model in which one omega value less than one is inferred and a second omega value is set to 1 (M1); a selection model in which a third omega value greater than one is also inferred (M2); a beta distributed model of omega with eight omega values between 0 and 1 (M7); and a beta distributed model including an extra parameter for omega greater than one (M8). The Aikaike Information Criterion and appropriate likelihood ratio tests were used to infer which model best fit the data. PAML removed several codon positions due to ambiguous nucleotides and due to the stop codon at position 481 found in all samples from one clade of the Gabon 2001–05 outbreaks [27]. Bayes empirical Bayes estimates from PAML were used to infer positive selection.

### Prediction of disorder

The protein sequence of the glycoprotein (UniProt ID: Q05320) from Ebola virus [49] and the secreted glycoprotein from GenBank accession AY142960 were used in the disorder prediction program VSL2 [50] to infer ordered vs disordered regions of the spike and secreted glycoproteins, respectively. Scores greater than 0.5 indicate predicted disorder. VSL2 uses multiple neural network predictors based upon amino acid sequence and physical attributes to address different flavors of disorder, as well as, differences between short and long disordered regions [51, 52]. This predictor achieves well-balanced accuracy for both short (less than 30 residues) and long (more than 30 residues) disordered regions.

## Supporting Information

S1 Fig Unrooted phylogeny of EBOV glycoprotein gene with GenBank accessions. Scale bar indicates number of substitutions per site.
(PDF)
