# Supplementary figures and images for: New Perspectives on Ebola Virus Evolution
Source: PLoS One. 2016 Aug 1;11(8):e0160410. doi: 10.1371/journal.pone.0160410 (PMC4968807; doi:10.1371/journal.pone.0160410)

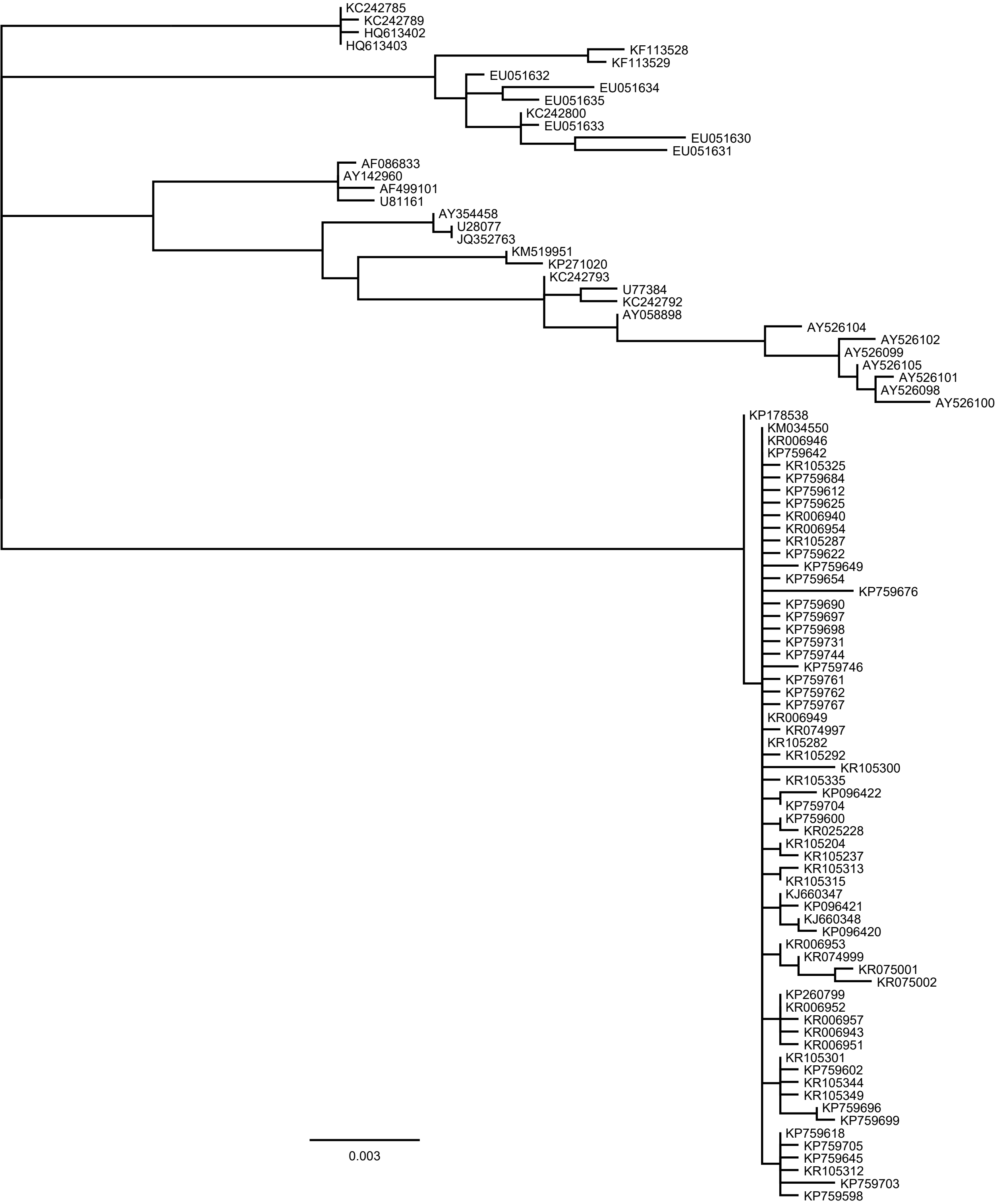

Supplement: S1 Fig — Scale bar indicates number of substitutions per site. (PDF) [file pone.0160410.s001.pdf]
